# Upregulation of PEDF Predicts a Poor Prognosis and Promotes Esophageal Squamous Cell Carcinoma Progression by Modulating the MAPK/ERK Signaling Pathway

**DOI:** 10.3389/fonc.2021.625612

**Published:** 2021-02-26

**Authors:** Zui Chen, Di Che, Xiaoqiong Gu, Jiamin Lin, Jing Deng, Ping Jiang, Kaixiong Xu, Banglao Xu, Ting Zhang

**Affiliations:** ^1^ Department of Laboratory Medicine, Guangzhou First People’s Hospital, School of Medicine, South China University of Technology, Guangzhou, China; ^2^ Department of Clinical Biological Resource Bank, Guangzhou Institute of Pediatrics, Guangzhou Women and Children’s Medical Center, Guangzhou Medical University, Guangzhou, China

**Keywords:** pigment epithelium-derived factor, esophageal squamous cell carcinoma, prognosis, MAPK/ERK, metastasis

## Abstract

Invasion and metastasis represent the primary causes of therapeutic failure in patients diagnosed with esophageal squamous cell carcinoma (ESCC). The lack of effective treatment strategies for metastatic ESCC is the major cause of the low survival rate. Therefore, it is crucial to understand the molecular mechanisms underlying ESCC metastasis and identify potential biomarkers for targeted therapy. Herein, we reported that PEDF is significantly correlated with tumor cell invasion and metastasis in ESCC. The high expression of PEDF is an independent unfavorable prognostic factor for ESCC patients’ overall survival (OS). We successfully developed and verified a nomogram to predict the preoperative OS of ESCC patients, and the actual and nomogram-predicted 1-, 3-, and 5-year survival rates had good consistency. The receiver operating characteristic (ROC) curve showed that the area under the curve (AUC) values for 1-, 3- and 5- survival were 0.764, 0.871, and 0.91, respectively. Overexpression of PEDF significantly promoted the migration and invasion of ESCC cells *in vitro*, while silencing PEDF yielded the opposite effects. Elevated levels of PEDF altered the expression of proteins involved in epithelial–mesenchymal transition (EMT), as indicated by the upregulation of N-cadherin and the downregulation of *α*-catenin and E-cadherin in ESCC cells. Mechanistically, PEDF promoted tumor cell motility and EMT by activating the MAPK/ERK signaling pathway. In conclusion, our results reveal that PEDF is involved in ESCC metastasis and could act as a prognostic factor for ESCC. Our research provides a fresh perspective into the mechanism of ESCC metastasis.

## Introduction

Esophageal carcinoma ranked sixth among the causes of cancer-related death globally in 2018, and its incidence has rapidly increased in recent decades. The prevalence and mortality rate of esophageal carcinoma in China rank fifth worldwide, but China accounts for approximately half of all new cases and deaths (1, 2). Pathologically, there are two main forms of esophageal carcinoma: adenocarcinoma and squamous cell carcinoma. However, there are also regional differences between these two histological types. Esophageal squamous cell carcinoma (ESCC) is the most common histologic type of esophageal cancer worldwide. ESCC patients often already exhibit advanced metastasis at the time of diagnosis (3). The prognosis of ESCC patients is generally poor and is dependent on the existence of metastases (4, 5). Thus, therapeutic approaches are most likely dependent on the existence of tumor metastases, the genetic composition and the tumor subtype (6). Hence, more detailed works in the future need to effectively explore the mechanism of ESCC at the molecular level and develop improved diagnostic and therapeutic agents.

Metastasis is a multistep process in which cancer cells spread from the primary site to a local or distant location by acquiring malignant characteristics (7, 8). Epithelial–mesenchymal transition (EMT) is a crucial step in tumor metastasis and is correlated with a high mortality rate in cancer patients; during EMT, benign cancer cells transform from an inactive state to an extremely active and invasive state (9). Previous studies have shown that EMT participates in the acquisition of cancer stem cell phenotypes (10). In addition, EMT includes several special processes in which epithelial cells disrupt cell–cell contact, cell–matrix adhesion, and apical–basal polarity and concomitantly acquire a phenotype that leads to invasion and migration (11, 12). The changes in EMT are accompanied by alterations in the expression of EMT-related proteins. Hallmarks of EMT include downregulation of epithelial markers (such as *α*-catenin and E-cadherin), upregulation of mesenchymal markers (such as N-cadherin), and induction of focal adhesion turnover (13, 14). When metastasis occurs in ESCC, cancer cells obtain mesenchymal characteristics and exhibit increased N-cadherin expression and decreased E-cadherin expression (15, 16). EMT is tightly related to ESCC invasion and metastasis; the promotion of ESCC cell EMT could dramatically increase the likelihood of ESCC metastasis (17).

Pigment epithelium-derived factor (PEDF) is a 50-kDa secreted endogenous glycoprotein encoded by the serine protease inhibitor-clade F1 (SERPINF1) gene, a member of the serine protease inhibitor superfamily; it was first isolated from human fetal retina pigment epithelial cells (18). It has been established that PEDF plays an important role in tumor angiogenesis, growth, and migration. Therefore, PEDF has attracted significant attention in the field of cancer therapeutics (19). Indeed, many previous studies have demonstrated that PEDF inhibits the proliferation and migration of nasopharyngeal carcinoma (20), pancreatic cancer (21), glioma (22), melanoma (23), and breast cancer (24) cells. Nevertheless, in a few cases, PEDF can promote the occurrence and development of tumors. PEDF drives glioma stem cell proliferation and self-renewal (25). PEDF is also significantly associated with hepatocellular carcinoma progression and metastasis (26). Recently, Tang et al. reported that PEDF promotes esophageal cancer cell growth (27). However, the molecular mechanism by which PEDF functions in the tumorigenesis and clinical progression of ESCC remains elusive.

In the present study, we determined that PEDF was positively related to tumor metastasis and an adverse prognosis through various investigations. We established a nomogram that incorporates PEDF, age, gender, tumor-node-metastasis (TNM) stage and could be conveniently used to facilitate the preoperative prediction of survival time of individual patients with ESCC. Moreover, we demonstrated for the first time the relationship between the abnormal expression of PEDF and the progression of ESCC and reported that PEDF augments ESCC cell migration, invasion, and EMT. We also revealed the underlying molecular mechanisms by which PEDF causes tumor metastasis: PEDF activates the MAPK/ERK pathway, which consequently promotes EMT. These findings demonstrate that PEDF could promote ESCC progression by positively regulating the MAPK/ERK pathway, thus providing new insights into the metastasis of ESCC and a reference for developing clinical intervention strategies for ESCC.

## Materials and Methods

### Data Collection and Preprocessing

The mRNA expression datasets were downloaded from the GEO (ID: GSE19472) and TCGA. First of all, one comparison (invasion cells *vs*. non-invasion cells) was conducted in this array. Differentially expressed mRNAs with *p*-value <0.05 and fold change (FC) >average (log FC) + 2 ∗ SD (log FC) was considered significantly different. Normalized log-transformed array data and enrichment analyses for the transcriptome and GEO data sets were conducted by R software (version 4.0.0). The genomic and clinical data of esophageal carcinoma were downloaded from TCGA and the RNA sequencing data from Illumina platform.

### Cell Lines and Culture

All ESCC cells including KYSE140 and KYSE510, were cultured using RPIM 1640 medium (Gibco, Beijing, China) supplemented with 10% fetal bovine serum (FBS) (Gibco, Montevideo, Uruguay), 100 U ml^−1^ penicillin, and 100 mg ml^−1^ streptomycin. All ESCC cells were incubated at 37°C containing a 5% CO_2_ environment.

### siRNA and Plasmid Transfection

For transient knockdown or overexpression of PEDF, ESCC cells were plated at 6 × 10^5^ cells in 6-well plates (Corning, Michigan, USA) with 2 ml medium. All cells were then transfected with specific siRNA duplexes or plasmid using Lipofectamine 3000 (Invitrogen, Shanghai, USA) and serum-free medium according to the manufacturer’s instructions. RiboBio (Guangzhou, China) made the siRNAs whose sequences target human PEDF: 5′-CCCGGATCGTCTTTGAGAA-3′ (si1-PEDF), 5′-GAACAGAATCCATCATTCA-3′ (si2-PEDF), 5′-TCACCAGACTTTAGCAAGA-3′ (si3-PEDF), and 5′-CATTAATGTCGGACAAC-3′ for the negative control sequence.

### RT-qPCR

The total RNA of ESCC cell lines treated with Trizol reagent (Invitrogen, Shanghai, USA) or Ultrapure RNA Kit (KangWei, Beijing, China) and cDNA synthesis by using the PrimeScript RT reagent Kit (TaKaRa Bio Inc, Dalian, China). The quantitative real-time PCR assay for PEDF was carried out using the SYBR Premix Ex Taq (TaKaRa Bio Inc, Dalian, China). GAPDH was served as normalizing the expression of mRNA, respectively. All results were repeated three or more times. The fold changes were calculated using the 2-ΔΔCt method. The sequences of real-time PCR primers for *β*-actin and PEDF were as follows: *β*-actin forward: 5′-GCACTCTTCCAGCCTTCCTT-3′ and *β*-actin reverse: 5′-GTTGGCGTACAGGTCTTTGC-3′, and PEDF forward 5′-CAGAAGAACCTCAAGAGRGCC-3′ and PEDF reverse 5′-CTTCATCCAAGTAGAAATCC-3′, respectively.

### Western Blot Analysis

Western blotting analyses were performed by using standard methods. The cells were washed three times with PBS and collected by the RIPA lysis buffer containing protease inhibitors (Beyotime Biotechnology, Shanghai, China). Each sample was added the same quality protein, and volume was loaded onto 10% SDS–polyacrylamide gel electrophoresis gels. The proteins were transferred to PVDF membranes (Millipore, MA, USA) and blocked 1 h with QuickBlock Blocking Buffer (Beyotime Biotechnology, Shanghai, China) at room temperature. The membrane was incubated with primary antibody at 4°C overnight and then with the corresponding peroxidase-conjugated secondary antibody 1–2 h at 37°C, detected with electrochemiluminescence reagent (Affinity Biosciences, Shanghai, USA). The same membrane was stripped and soaked in the anti-GAPDH antibody to control for differences in protein loading.

The primarily used antibodies included anti-E-cadherin (610181), anti-*α*-catenin(610193) and anti-N-cadherin (610921) from BD Pharmingen (San Diego, CA, USA); anti-p-ERK1/2(#9102), anti-ERK1/2(#4370), anti-ATK(#4691S), anti-pAKT(#4060S) and anti-GAPDH(2118S) from Cell Signaling Technology (Danvers, MA, USA); anti-PEDF(MAB1059) from Millipore (Millipore, MA, USA).

### Wound Healing Assays

ESCC cells were seeded into six-well plates at a density of 4 × 10^5^ cells/well and allowed to grow to 80–90% confluent in complete medium at 37°C. Then all cells were starved for 16 h in a serum-free medium (Gibco, Beijing, China). The monolayers were disrupted by scraping them with a sterile 10-ul tip and floating cells were removed by washing with PBS. The cells’ migration progress into the wound was photographed by an inverted microscope (Leica DMI4000B, Wetzlar, Germany) at 0, 24, and 48 h. Each dish was measured three times at each wound, and the average value was counted.

### Migration and Invasion Assays

A total of 8 × 10^4^ (for KYSE140) or 4× 10^4^ (for KYSE510) cells in 200 μl of serum-free RPIM 1640 were transferred to the superstratum chamber inserts with 8 μm pore size fibronectin-coated polycarbonate membrane (Corning, Corning, NY, USA) and 750 μl of RPIM 1640 containing 20% FBS was added into the chamber below. KYSE140 cells were incubated for 36 h, respectively, while KYSE510 cells were incubated for 24 h, and then the upper chamber was soaked in 4% PFA Fix Solution (JingXin, Guangzhou, China) for 20 min with removing the cells on the higher surface of the membrane, after the sample was dried stained with crystal violet for 15 min, and then the number of cells on the membrane was counted by inverted microscope (×100 magnification). All experiments were repeated three times unless indicated otherwise. Cell invasion assays were performed as the migration assay, except that the upper membrane was coated with Matrigel before the step seeds cell 4 h (BD Biosciences, Franklin Lakes, NJ, USA).

### Patients and Samples

Human tissue specimen microarrays were purchased from Shanghai Outdo Biotech CO, LTD. ESCC specimens and normal-looking squamous mucosa specimens were used to quantify PEDF expression. All patients were definite with ESCC through clinical and pathological examinations. Clinicopathological information was derived from ESCC patient clinical, pathological, and outcomes data. Our research was approved by the Shanghai Outdo Biotech Company ethics committee.

### Ethics Approval and Consent to Participate

The human tissue specimen research was approved by the institutional ethics committee of Shanghai Outdo Biotech Co, Shanghai, China (Exp. number: YB M-05-02).

### Immunohistochemistry

Tissue microarrays that contained 114 ESCC tissues were created following the routine protocols. The paraffin-embedded tissues were cut into 4 μm thickness sections, and the procedures of immunohistochemical staining of PEDF were followed. After being baked at 65°C for 1–2 h, dewaxed in dimethyl benzene and hydration in a gradient of alcohol concentrations, the tissues were subjected to citrate antigen recovery buffer 125°C for 10 min. Endogenous peroxidase activity and non-specific binding of antibodies were blocked by 3% H_2_O_2_ and 1% goat serum albumin, respectively. The samples were then probed with primary antibodies against PEDF (Millipore, MA, USA) at 4°C for 18 h. The slides were incubated with appropriate secondary antibodies to bind the primary antibodies, followed by an avidin–biotin technique with diaminobenzidine (DAB) visualization, and counterstaining with hematoxylin. The immunostained tissue microarray slides were photographed by Leica SCN400 slide scanner (Meyer Instruments, Houston, TX, USA). The staining intensity of PEDF was scored and graded from 0 to 3: 0 (no expression), 1 (weak expression), 2 (moderate expression), 3 (strong expression), and every tumor was scored on a scale of 0–4 according to the percentage of cells with cell staining: 0 (0%), 1 (1–25%), 2 (26–50%), 3 (51–75%%), and 4 (≥76%). The final total histological score (score ranges from 0 to 12) was determined by calculating as intensity score × percentage score: 0 to 5 was regarded as low expression, and ≥6 was considered high expression.

### Statistical Analysis

All the assays and experiments were performed at least thrice with triplicate repeats. All results were expressed as the mean ± standard deviation (S.D.). One-way analysis of variance (ANOVA) or Student’s test was used to perform statistical differences. All statistical analyses were performed using SPSS 24(Chicago, IL, USA) or GraphPad Prism 8.0 (GraphPad Software, La Jolla, CA, USA). The *p*-value of less than 0.05 was indicated as statistically significant.

## Results

### PEDF mRNA Expression Is Upregulated in Metastatic Esophageal Carcinoma Cell Lines and Tissues

**Figure 1:**
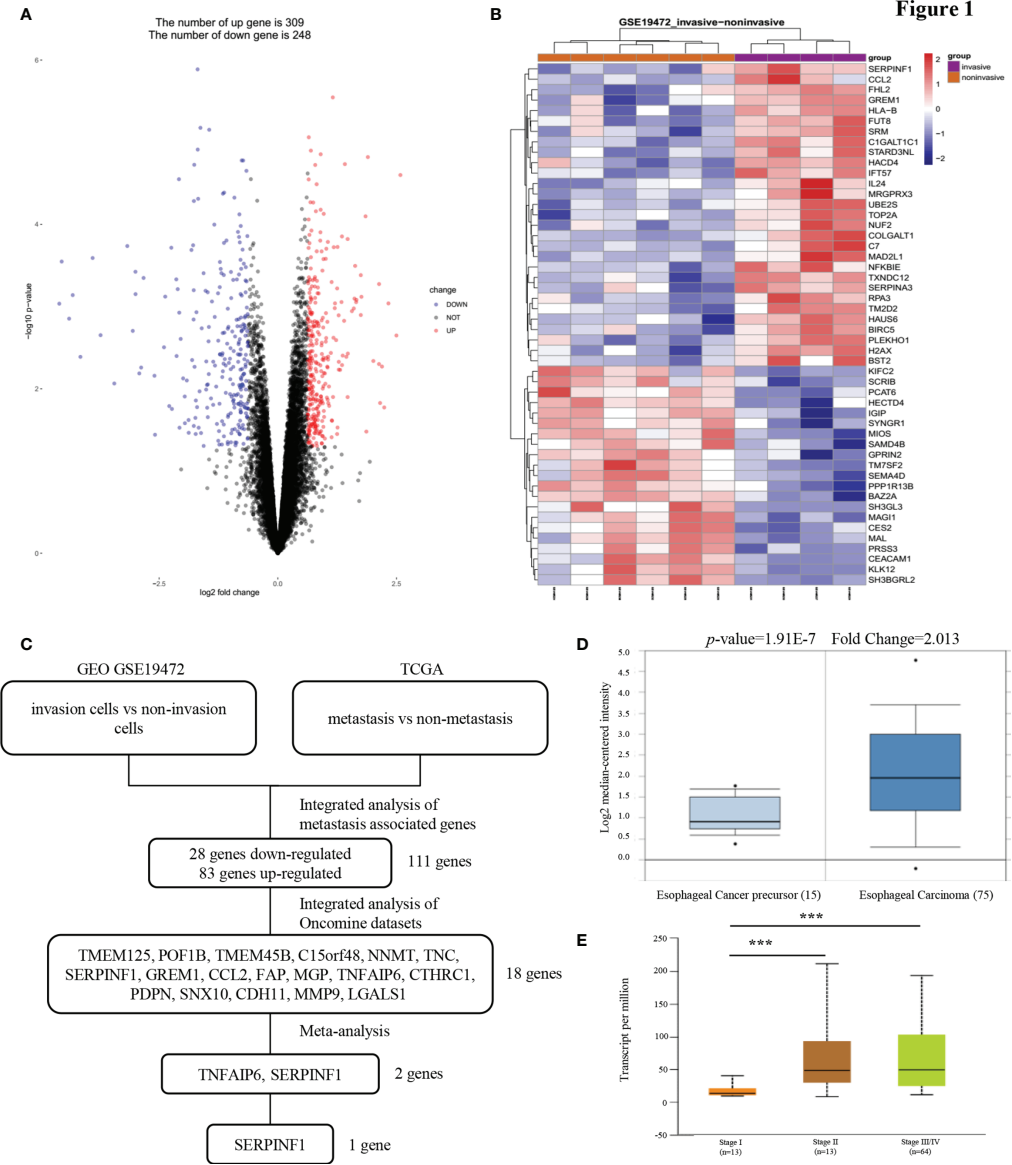
PEDF expression is upregulated in esophageal carcinoma invasion cells. **(A)** A Pearson test was used to analyze genes differentially expressed in ESCC. **(B)** The cluster heat maps showing the 50 different expressed mRNAs in four lines of ESCC model invasion cells and corresponding six lines of ESCC model non-invasion cells. The blue and red stripes represent downregulated and upregulated mRNAs respectively. **(C)** Microarray analysis was performed to identify metastasis associated genes. Expression levels were analyzed in GEO (invasion cells *vs* non-invasion cells) and TCGA (metastasis *vs* non-metastasis). In order to rule out effects of precancerous lesions, esophageal cancer precursor genes and esophageal carcinoma genes were identified in Oncomine microarray dataset, as shown in the flowchart. These data were integrated with meta-analysis to identify the potential esophageal carcinoma metastasis associated genes (two genes). **(D)** Representative PEDF mRNA expression in esophageal carcinoma tissues *versus* esophageal cancer precursor samples from the Kim Esophagus dataset. **(E)** The significant association between clinic stages and PEDF expression levels. ***P < 0.001.

To identify mRNAs that could predict the metastasis and prognosis of ESCC, we analyzed mRNA profiling arrays derived from the Gene Expression Omnibus (GEO) (ID: GSE19472) (**Figures 1A, B**). One comparison (invasive cells *vs* non-invasive cells) was conducted in this mRNA profiling array to identify mRNAs were differentially expressed in non-invasive cells *versus* invasive cells. Transcript changes with *p <*0.05 and fold change (FC) >average (log FC) + 2 ∗ SD (log FC) were considered significant. The cell migration and invasion capabilities always correlate with cancer metastasis. In our study of the reliability of the analysis, we found that the expression of only 111 genes was consistent between the GEO and The Cancer Genome Atlas (TCGA) databases. To narrow the possible candidate genes, the expression of these 111 genes was also evaluated by using Oncomine. We identified 18 possible genes that were associated with ESCC metastasis. After scrutinizing the literature, we found two genes that promote tumor metastasis that are less reported among the 18 genes involved in tumor metastasis regulation of ESCC (**Figure 1C**). We found that the expression of PEDF in esophageal carcinoma was different from that in other cancers and that the expression of PEDF in esophageal cancer precursor samples was lower than that in esophageal carcinoma samples (**Figures 1D** and **S1A**). Interestingly, the expression of PEDF was significantly associated with the clinical stage (stage I *vs*. stage II/stage III, *p* < 0.001) of ESCC patients (**Figure 1E**). We also analyzed the expression data of PEDF in normal esophageal tissues and ESCC tissues in the TCGA database. No obvious difference in the relative levels of PEDF was found between ESCC samples and normal esophageal tissues (*p* = 0.47) in either database (**Figure S2A**). PEDF was significantly positively correlated with the number of lymph node metastases (N0 vs N1/N2, *p* < 0.001) (**Figure S2B**). These observations suggest that high PEDF expression may play a key role in ESCC metastasis and may be a novel prognostic factor for ESCC patients.

### Overexpression of PEDF Is Correlated With an Unfavorable Prognosis in ESCC Patients

**Figure 2:**
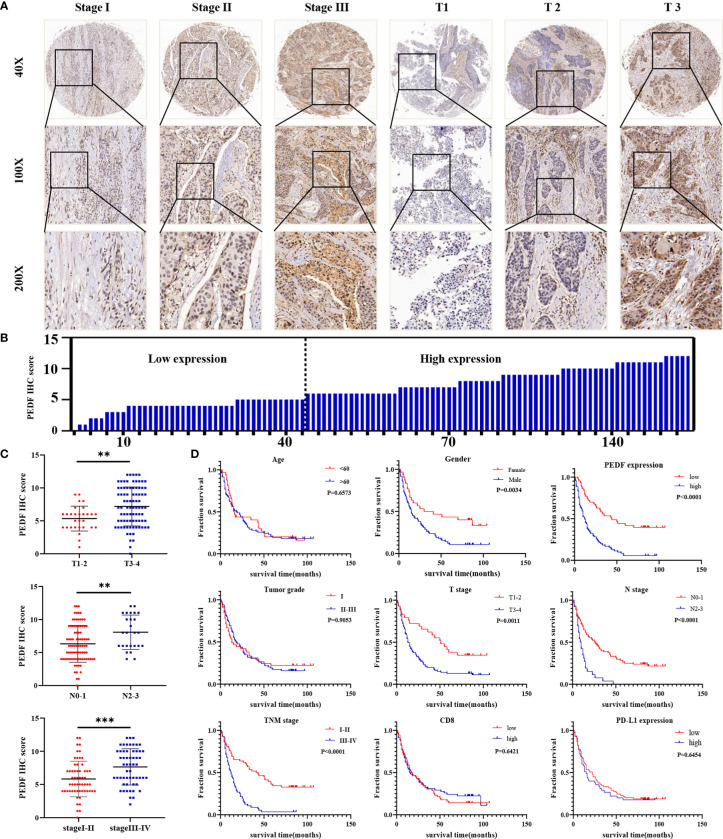
PEDF is abnormally expressed in advanced ESCC patients and related to clinical indicators. **(A)** Levels of PEDF protein expression in ESCC tissue chip are shown under both low and high magnifications. **(B)** The immunohistochemical score was calculated for each ESCC samples. Tumors with an expression level lower than six points are considered the low expression, while six points or more are considered high expression. **(C)** Graph showing the IHC staining scores and pathological clinical stage in 114 ESCC patients. **P < 0.01, ***P < 0.001. **(D)** Kaplan–Meier curve analysis revealed that ESCC patients in high PEDF expression had shorter survival time than that of ESCC patients with low expression of PEDF. The survival time was significantly associated with gender, T stage, N stage, TNM stage, but not with age, tumor grade, CD8, PD-L1.

To investigate the clinical value of increased expression of PEDF in ESCC, we used a group of 114 ESCC tissues on tissue microarrays and probed the expression of PEDF by IHC (**Figures 2A, B**). Furthermore, we analyzed the relationship between the clinicopathological characteristics of ESCC patients and PEDF expression. We found a positive and significant correlation between PEDF expression and N stage (*p* = 0.01), CD8 (*p* = 0.006), and TNM stage (*p* =0.002). However, other clinicopathological factors, including T stage, age, gender, tumor grade, and PD-L1, were not correlated with PEDF expression (**Table 1**). These results also revealed that there might be a positive correlation between PEDF expression and T stage (*p* = 0.002), N stage (*p* = 0.005), and TNM stage (*p* < 0.001) (**Figure 2C**). Kaplan–Meier survival curves showed that female patients (*p* = 0.0034), patients with low expression of PEDF (cut-off score <6) (*p* < 0.001), patients with N0–1 stage disease (*p* < 0.001) and patients with TNM stage I/II disease (*p* < 0.001) had significantly longer survival times than male patients, patients with overexpression of PEDF, patients with N2–3 stage disease and patients with TNM stage III/IV disease, respectively (**Figure 2D**). Cox proportional hazard regression analyses revealed that high PEDF expression was an independent predictor of unfavorable prognosis in ESCC patients (hazard ratio [HR] = 3.173, *p* < 0.001) (**Table 2**). Altogether, these results suggest that PEDF could be an ESCC metastasis promoter and that overexpression of PEDF is associated with a poor prognosis in patients with ESCC.

**Table 1:** Association between the expression of PEDF and main clinical characteristics in 114 ESCC patients.

Characteristic		All cases	PEDF		Chi-square	P value
			low	high		
All cases		114	43	71		
Age	<60	32	16	16	2.865	0.132
	≥60	82	27	55		
Gender	Female	30	14	16	1.387	0.239
	Male	84	29	55		
Grade	I	45	19	26	0.642	0.473
	II–III	69	24	45		
T stage	T1–2	29	14	15	1.854	0.190
	T3–4	85	29	56		
N stage	N0–1	88	39	49	7.152	0.010^**^
	N2–3	26	4	22		
TNM stage	I–II	58	30	28	9.858	0.002^**^
	II–IV	56	13	43		
PD-L1	Low	69	24	45	0.641	0.423
	high	45	19	26		
CD8	Low	56	14	42	7.580	0.006^**^
	high	58	29	56		

*P < 0.05, **P < 0.01.Statistical significance (p < 0.05) is shown in bold.

**Table 2:** Univariate and multivariate Cox hazards analyses of different parameters for OS in 114 ESCC patients.

Clinical variables	Univariate analysis		P	Multivariate analysis		P
	HR	95% CI		HR	95% CI	
Age (<60 vs. ≥60)	1.095	0.696–1.724	0.695			
Gender (Female vs. Male)	2.177	1.295–3.658	**0.003**	1.769	1.039–3.013	**0.036**
Grade (I vs. II/III)	1.025	0.673–1.560	0.909			
T stage (T1/2 vs. T3/4)	2.245	1.345–3.748	**0.002**			
N stage (N0–1 vs. N2–3)	2.850	1.759–4.618	**<0.001**			
TNM stage (I/II vs. III/IV)	3.038	1.952–4.730	**<0.001**	2.200	1.379–3.510	**<0.001**
PEDF (low vs. high)	3.173	1.982–5.081	**<0.001**	2.494	1.528–4.070	**<0.001**
PD-L1 (low *vs*. high)	1.101	0.727–1.669	0.649			
CD8 (low *vs*. high)	0.885	0.589–1.331	0.558			

CI, confidence interval; HR hazard ratio. Statistical significance (p < 0.05) is shown in bold.

**Figure 3:**
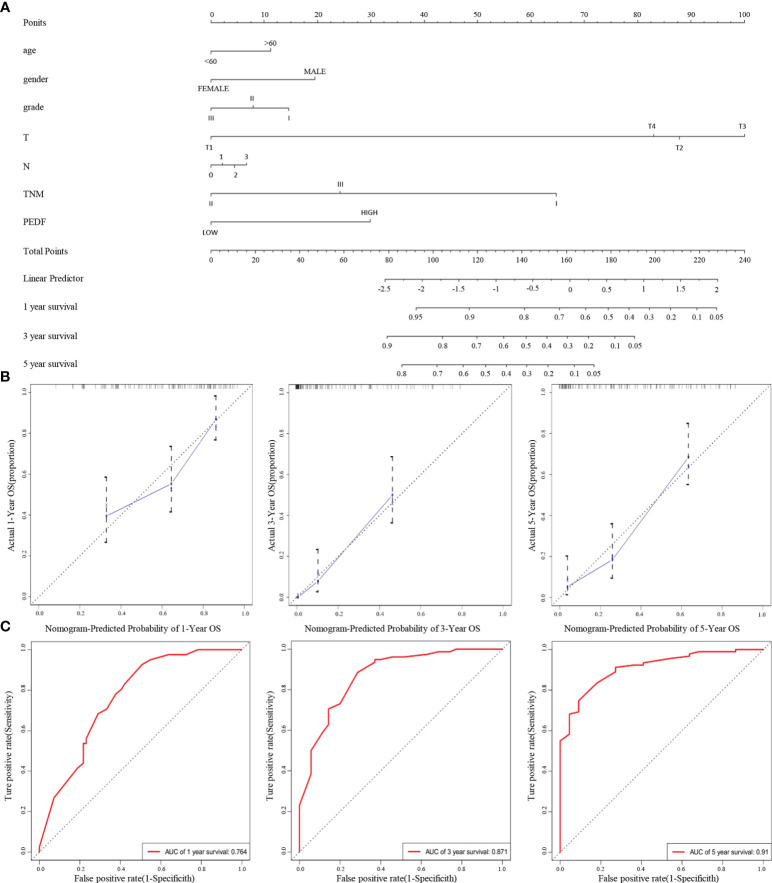
A nomogram prediction model was constructed based on the expression of PEDF in ESCC. **(A)** Nomogram system for the 1-, 3-, and 5- year survival rate prediction. The nomogram prediction system was a novel model to estimate OS based on related factors (tumor grade, T stage, N stage, TNM), patient-specific factors (age, gender) and PEDF expression. **(B)** Calibration plots for predicting for ESCC OS at 1-, 3-, and 5-year. The blue dotted line indicates the ideal nomogram; blue X indicates the bootstrap-corrected estimates; vertical bars indicate the 95% CIs. **(C)** ROC curves of the 1-, 3-, and 5-year nomograms of ESCC patients. The red bars represent a new nomogram predicted OS, whereas the black bars represent the TNM stage predicted OS.

Furthermore, according to the multivariate regression analysis, we established a novel nomogram model, including PEDF expression in combination with age, gender, T stage, N stage, TNM stage, and tumour grade, to predict the individual ESCC patients of 1-, 3-, and 5-year overall survival (OS) rates of individual ESCC patients (**Figure 3A**). The calibration plots of the nomogram showed good agreement between the actual and nomogram-predicted 1-, 3- and 5-year survival rates (**Figure 3B**). The ROC curves for the nomogram indicated that the AUC values for 1-, 3- and 5-year OS were 0.764, 0.871 and 0.91, respectively (**Figure 3C**). These data reveal that the combination of PEDF expression assessment with TNM staging could establish a better predictive scale for ESCC patient prognosis.

### PEDF Promotes ESCC Cell Migration, Invasion, and EMT

**Figure 4:**
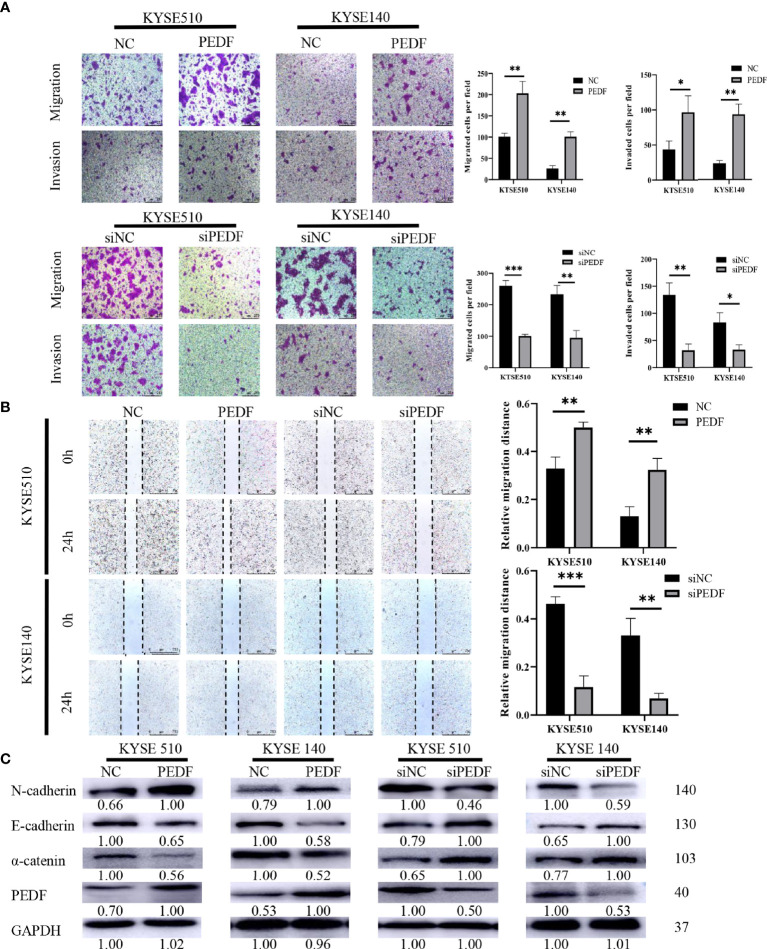
PEDF promotes ESCC cell migration, invasion, and EMT. **(A)** The KYSE510 and KYSE140 cellular migration and invasion capability were assessed by transwell migration and Boyden chamber invasion assays. The mean numbers of migrating or invading cells in the field were calculated by mean ± SD from three-time different experiments. Magnification for ×100. *P < 0.05, **P < 0.01, ***P < 0.001. **(B)** KYSE510 and KYSE140 cells and appropriate controls were used in a wound-healing assay. Scale bar = 100 μm. (n = 3 each). Data are means ± S.E.M. *P < 0.05, **P < 0.01. **(C)** The expression of *α*-catenin, N-cadherin, and E-cadherin were probed by western blotting in overexpress of PEDF KYSE510 and KYSE140 cells, and PEDF inhibition KYSE510 and KYSE140 cells, and the corresponding control cells.

To determine the role of PEDF in ESCC metastasis, we constructed a PEDF overexpression plasmid and designed three short-interfering RNA (siRNA) sequences to target PEDF. We analyzed the qRT-PCR results and found that PEDF expression was significantly upregulated in ESCC cells transfected with the plasmid and that PEDF expression was remarkably downregulated in ESCC cells transfected with the siRNAs (**Figure S3**). Among the three siRNAs, si1-PEDF exhibited the highest interference efficiency in ESCC cells. Therefore, it was selected for further functional study. PEDF was overexpressed by using plasmid or knocked down by using siRNA in KYSE140 and KYSE510 cells. Wound healing assays, Transwell migration assays, and Boyden chamber assays were performed to assess whether PEDF influences ESCC cellular mobility. These experiments demonstrated that the migration and invasion capabilities of KYSE140-PEDF and KYSE510-PEDF cells, which overexpressed PEDF, were strengthened compared to those of control cells. In contrast, the migration and invasion capabilities of KYSE140-siPEDF and KYSE510-siPEDF knockdown cells were decreased compared with those of the negative control siRNA (si-NC)-transfected cells (**Figures 4A, B**). These results suggest that PEDF has cancer-promoting effects in ESCC cells.

Many studies have demonstrated that the process of cancer cell migration and invasion is closely linked to EMT, the process by which epithelial cells transform into mesenchymal cells by losing their polarity and adhesion capabilities and by obtaining invasive and migratory abilities (14). Thus, we measured the expression of EMT-related markers by Western blotting, and the results demonstrated that PEDF-overexpressing KYSE140 and KYSE510 cells exhibited a striking decrease in the expression of epithelial markers (E-cadherin and *α*-catenin) and an increase in the mesenchymal marker (N-cadherin) in comparison with that in the corresponding control cells. Consistent with the results in PEDF-overexpressing cells, we observed that silencing PEDF with siRNA increased E-cadherin and *α*-catenin expression but decreased N-cadherin expression in both KYSE140-siPEDF and KYSE510-siPEDF cells compared with si-NC cells (**Figure 4C**). Altogether, these results suggest that PEDF promotes cell motility and invasiveness by driving EMT in ESCC cells.

### MAPK/ERK Signaling Is Required for PEDF Mediated EMT

**Figure 5:**
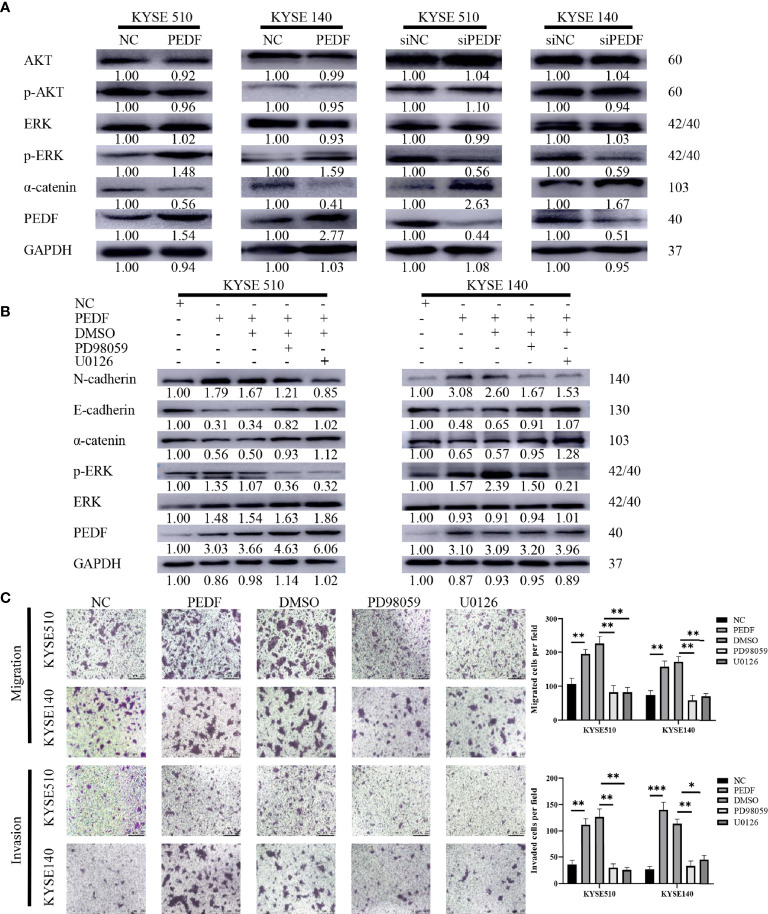
MAPK/ERK signaling pathway is required for PEDF mediated EMT. **(A)** The expressions of AKT, p-AKT, a-catenin, ERK1/2, p-ERK1/2 and PEDF were probed by western blotting in the overexpression of PEDF KYSE510 and KYSE140 cells, and PEDF knockdown KYSE510 and KYSE140 cells, and the corresponding control cells. GAPDH was a protein loading control. **(B)** Western blotting indicated that p-ERK1/2 inhibitor PD98059 and U0126 inhibited PEDF induced the activation of MAPK/ERK signaling pathway, as well as the EMT-related characterizations in KYSE510 and KYSE140 cells. Cells were treated with MAPK/ERK signaling agonist PD98059 or U0126 for 24 h, and then performed WB assay. P < 0.05, relative to PD98059 or U0126 treatment of the same cell type as controls. **(C)** The cell invasion and migration were evaluated after ERK1/2 inhibitor treatment for 24 h. Magnification for × 100. *P < 0.05, **P < 0.01, ***P < 0.001.

The large-scale genome sequencing results in the TCGA indicated that the ERK and phosphatidyl-inositol-3-kinase (PI3K) signaling pathways play key roles in the tumorigenesis and progression of ESCC (28, 29). Moreover, PEDF has been recently reported to regulate the MAPK/ERK and PI3K/AKT signaling pathways (30–33). Therefore, we sought to examine the potential role of the PI3K/AKT and MAPK/ERK pathways in the PEDF-induced EMT of ESCC cells. Surprisingly, PEDF overexpression did not significantly influence AKT expression or phosphorylated AKT expression but upregulated the expression of phosphorylated ERK1/2, suggesting that the activation of MAPK/ERK signaling by ERK1/2 phosphorylation plays a special role in promoting tumor migration and invasion induced by PEDF (**Figure 5A**). To validate our experimental results, we used the ERK inhibitors PD98059 and U0126 to block ERK1/2 phosphorylation in PEDF-overexpressing cells. The results showed that ERK1/2 inhibition by PD98059 and U0126 impaired the PEDF-induced migration, invasion, and EMT of KYSE140 and KYSE510 cells (**Figures 5B, C**). These results indicate that PEDF promotes esophageal cancer migration and invasion by activating the MAPK/ERK signaling pathway.

Altogether, these results indicate that PEDF strongly promotes ESCC metastasis. In short, overexpression of PEDF stimulates EMT, migration, and invasion through the MAPK/ERK pathway in ESCC.

## Discussion

Although metastasis is the primary cause of death in esophageal carcinoma patients, the underlying molecular mechanisms and biomarkers of esophageal carcinoma metastasis remain largely unclear (34, 35). PEDF (encoded by the SERPINF1 gene) was chosen as a key molecule not only because PEDF is significantly differentially expressed between malignant and benign esophageal cells according to mRNA array studies but also because the expression of PEDF in esophageal cancer is different from that in other cancers (**Figures S1A, B**). For example, PEDF has been verified as a tumor suppressor gene in nasopharyngeal carcinoma, breast cancer and other tumors, whereas the expression of PEDF in esophageal cancer precursors is significantly lower than that in esophageal cancer. This pattern of PEDF expression is unique to esophageal cancer and may indicate that PEDF has a certain cancer-promoting effect in esophageal cancer, but the specific function of PEDF in this context is still unclear. PEDF is considered to be a key molecule that regulates cell proliferation, migration, survival, and differentiation (36). According to reports, it acts as both a tumor promoter and a tumor suppressor by regulating multiple signaling pathways during tumorigenesis. Although PEDF has been shown to play a vital role in the metastasis of many types of tumors, there are few reports on how PEDF affects the progression of ESCC. Tang et al. previously reported that PEDF could promote esophageal tumor cell proliferation and apoptosis (27). In their study, they found that PEDF is overexpressed in esophageal carcinoma tissues and cells. In addition, they found that PEDF promotes esophageal cancer cell growth both *in vivo* and *in vitro*. In the present study, we focused on the role of PEDF as an enhancing factor rather than an initiating factor in ESCC progression. Thus, we investigated the associations between the different clinicopathological characteristics and the PEDF expression levels of ESCC patients. We assessed the clinical relevance of these findings and identified a positive correlation between PEDF expression and TNM stage in ESCC. We trained prediction models for ESCC using survival time as the outcome and PEDF data as a predictor. Moreover, we identified a new mechanism by which PEDF activates the MAPK/ERK signaling pathway to promote ESCC migration and invasion by inducing EMT. Our experiments showed that the overexpression of PEDF can promote the migration and invasion of esophageal cancer cells *in vitro*. In conclusion, in this preliminary study, we found that PEDF may be an important factor in ESCC metastasis.

In this study, we found that PEDF expression was lower in primary lesions than in metastatic lesions. Moreover, our multivariate analysis revealed that high PEDF levels and advanced TNM stage were crucial predictors of an unfavourable prognosis in ESCC patients. Overall, these results showed that overexpression of PEDF is related to the degree of malignancy and poor outcomes in ESCC. According to its already established oncogenic role in various kinds of carcinomas, PEDF tends to promote ESCC metastasis. We generated a predictive nomogram to predict the OS probability of individual ESCC patients based on PEDF expression, T stage, N stage, TNM stage, age, gender, and tumor grade. Calibration plots of the predictive nomogram revealed that the nomogram-predicted 3-year probability was similar to the actual OS rate, indicating that our system has high specificity, stability, accuracy, and sensitivity, which makes it convenient for clinical practice (37). The use of the TNM staging, the most commonly used indicator in prognosis and treatment, is still controversial because it is based on the limited anatomical range of tumors without the consideration of any other prognostic biomarkers (38). Therefore, our study integrated TNM staging and PEDF expression to estimate the OS of ESCC patients, and the 1-, 3-, and 5-year AUC values of the nomogram indicated that it was more accurate for predicting prognosis than the TNM stage (**Figure 3C**). The clinical patient data further verified the crucial role of PEDF in ESCC metastasis.

EMT, an essential biological process in tumor metastasis, includes the loss of the epithelial phenotype, the acquisition of the mesenchymal phenotype and the accumulation of collagen (39, 40). Previous studies have shown that downregulation of PEDF is associated with an increased incidence of EMT in breast and nasopharyngeal cancer tissues (20, 41). High PEDF expression in MD-231 breast cancer cells reduced cancer cell migration and fibronectin expression but did not affect the EMT phenotype (24). In the present research, we found that PEDF significantly promoted ESCC migration and invasion by driving EMT *in vitro*. Further *in vivo* study will be required to verify these *in vitro* experimental results. Moreover, PEDF overexpression reduced the expression of the epithelial markers *α*-catenin and E-cadherin and increased the expression of the interstitial marker N-cadherin, suggesting that PEDF may be a pivotal modulator involved in the EMT of ESCC cells. However, because PEDF plays complicated roles in various kinds of carcinomas and because cells undergoing EMT may share some molecular and morphological markers with the surrounding stromal cells (42), it is somewhat difficult to explain the details of the underlying mechanism by which PEDF positively or negatively regulates the EMT process in different cancers. PEDF has been shown to play contrasting roles in various tumors. The reason may be that tumors have significant heterogeneity, and tumors from different tissues may have different gene alterations.

The large-scale genome sequencing results from the TCGA indicate that the ERK and PI3K signaling pathways play key roles in the tumorigenesis and progression of ESCC (28, 29). Moreover, PEDF has been recently reported to regulate the MAPK/ERK and PI3K/AKT signaling pathways (30–33). Our work identified the MAPK/ERK signaling pathway plays a predominant role in PEDF-mediated ESCC progression. The MAPK/ERK signaling pathway has emerged as one of the most commonly dysregulated signaling pathways in cancer and has attracted extensive attention (43, 44). Previous studies have suggested that the MAPK/ERK signaling pathway is also involved in the migration and metastasis of cancer cells (45). Additionally, MAPK/ERK signaling inhibitors can suppress glioma cell migration and invasion (46, 47). A large body of evidence indicates that the MAPK/ERK signaling pathway is overactivated in the induction of EMT and the initiation of carcinogenesis (48–50). However, the potential upstream mechanism of ERK in esophageal cancer has only been demonstrated in a few papers (51). Consistent with our results, we found that PEDF might stimulate the progression of ESCC *via* the MAPK/ERK pathway because PEDF can increase the expression of phosphorylated ERK1/2 in esophageal carcinoma cells. Moreover, PD98059 and U0126, inhibitors of ERK1/2 phosphorylation, blocked the overexpression of PEDF, thus regulating EMT. We have not ruled out other signaling pathways that might also be involved in the process of PEDF-mediated EMT. Moreover, whether PEDF can phosphorylate and directly act on ERK remains to be validated in further research.

The reason why the cellular source of PEDF in ESCC is inconsistent with that in other carcinomas remains unclear. PEDF has been reported to participate in transcriptional regulation, hypoxia-mediated processes and posttranslational regulation (52). P53 mutation is the most dominant feature of ESCC (53). The findings of the sequenced genome analysis revealed that p63 is a transcription factor of the p53 gene family involved in ESCC carcinogenesis and progression. P63 amplification is more frequent and the expression of p63 is increased in ESCC samples compared with adjacent normal oesophagus and adenocarcinoma samples (28, 54–56). Alternative splicing leads to the generation of p63 isoforms harboring two different N-terminal (TA or ΔN) domains and three different C-terminal (*α*, *β*, and *γ*) domains (57). Δp63*α* is the predominant p63 isoform and mediates interactions between mesenchymal and epithelial cells in ESCC (58). PEDF is known to contain a p63 response element, a direct target of the p63 gene, in its promoter region. Overexpression of Δp63*α* has been shown to increase the expression of PEDF in tumors of various origins (59). However, it is unclear whether the abnormal expression of PEDF is caused by frequent Δp63*α* amplification in ESCC. Functional experiments are needed to further confirm this conjecture.

In conclusion, our results show that PEDF plays an oncogenic role by driving the migration and invasion of ESCC through the MAPK/ERK pathway. PEDF is a potential prognostic biomarker and new anti-metastatic target for ESCC.

## Data Availability Statement

The original contributions presented in the study are included in the article/**Supplementary Material**. Further inquiries can be directed to the corresponding authors.

## Ethics Statement

Written informed consent was obtained from the individuals for the publication of any potentially identifiable images or data included in this article.

## Author Contributions

TZ and BX conceived and designed this study. DC and JL extracted the information from the databases. ZC and KX performed experiments and prepared the manuscript. XG and PJ analyzed the data and processed specimens. JD and ZC carried out the cell culture. All authors contributed to the article and approved the submitted version.

## Funding

This study was supported by the National Natural Science Foundation of China (81702879), Key Sci-tech Research Project of Guangzhou Municipality, China (20190410237). The funding agencies had no role in study design, data collection, analysis, decision to publish, or manuscript preparation.

## Conflict of Interest

The authors declare that the research was conducted in the absence of any commercial or financial relationships that could be construed as a potential conflict of interest.
