# Supplementary material for: Upregulation of PEDF Predicts a Poor Prognosis and Promotes Esophageal Squamous Cell Carcinoma Progression by Modulating the MAPK/ERK Signaling Pathway
Source: Front Oncol. 2021 Feb 26;11:625612. doi: 10.3389/fonc.2021.625612 (PMC7953146; doi:10.3389/fonc.2021.625612)
Supplement: Supplementary file 1 [file DataSheet_1.pdf]

Supplementary Material

Supplementary Figures

A B

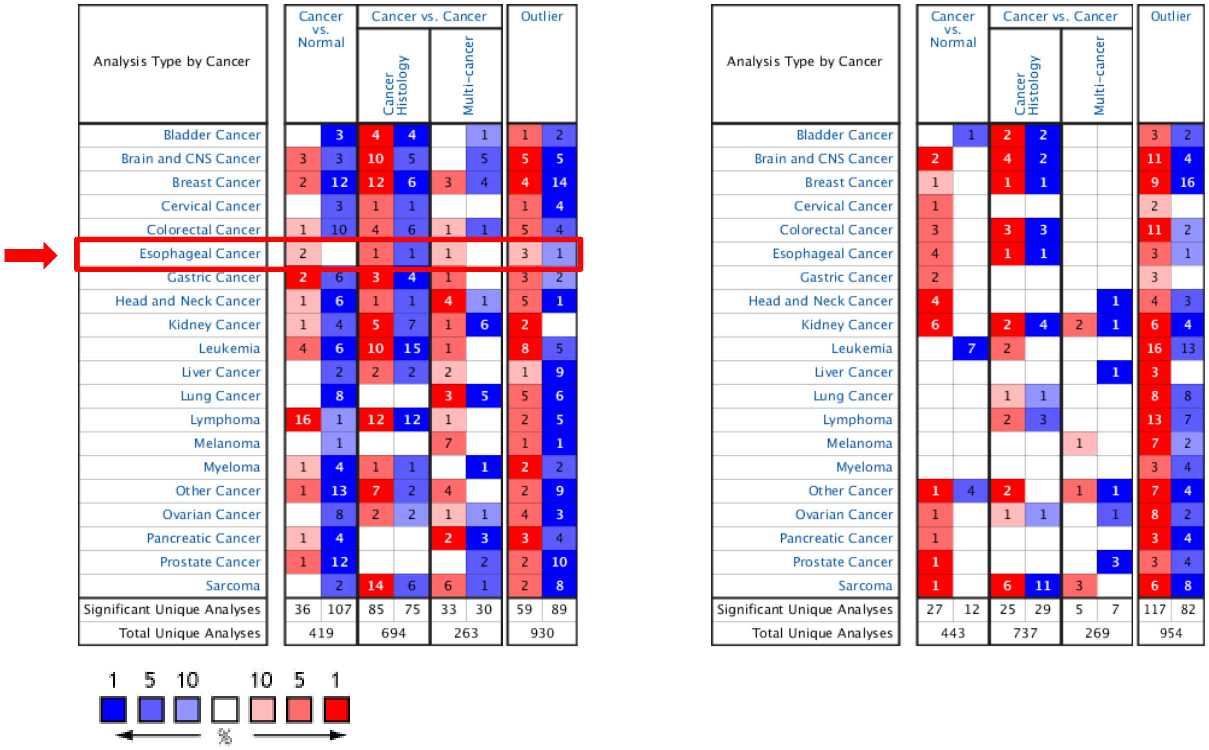

A

Analysis Type by Cancer	Cancer vs. Normal		Cancer vs. Cancer: Cancer Histology		Cancer vs. Cancer: Multi-cancer		Outlier	
Bladder Cancer		3	4	4		1	1	2
Brain and CNS Cancer	3	3	10	5		5	5	5
Breast Cancer	2	12	12	6	3	4	4	14
Cervical Cancer		3	1	1			1	4
Colorectal Cancer	1	10	4	6	1	1	5	4
Esophageal Cancer	2		1	1	1		3	1
Gastric Cancer	2	6	3	4	1		3	2
Head and Neck Cancer	1	6	1	1	4	1	5	1
Kidney Cancer	1	4	5	7	1	6	2	
Leukemia	4	6	10	15	1		8	5
Liver Cancer		2	2	2	2		1	9
Lung Cancer		8			3	5	5	6
Lymphoma	16	1	12	12	1		2	5
Melanoma		1			7		1	1
Myeloma	1	4	1	1		1	2	2
Other Cancer	1	13	7	2	4		2	9
Ovarian Cancer		8	2	2	1	1	4	3
Pancreatic Cancer	1	4			2	3	3	4
Prostate Cancer	1	12				2	2	10
Sarcoma		2	14	6	6	1	2	8
Significant Unique Analyses	36	107	85	75	33	30	59	89
Total Unique Analyses	419		694		263		930	

B

Analysis Type by Cancer	Cancer vs. Normal		Cancer vs. Cancer: Cancer Histology		Cancer vs. Cancer: Multi-cancer		Outlier	
Bladder Cancer		1	2	2			3	2
Brain and CNS Cancer	2		4	2			11	4
Breast Cancer	1		1	1			9	16
Cervical Cancer	1						2	
Colorectal Cancer	3		3	3			11	2
Esophageal Cancer	4		1	1			3	1
Gastric Cancer	2						3	
Head and Neck Cancer	4					1	4	3
Kidney Cancer	6		2	4	2	1	6	4
Leukemia		7	2				16	13
Liver Cancer						1	3	
Lung Cancer			1	1			8	8
Lymphoma			2	3			13	7
Melanoma					1		7	2
Myeloma							3	4
Other Cancer	1	4	2		1	1	7	4
Ovarian Cancer	1		1	1		1	8	2
Pancreatic Cancer	1						3	4
Prostate Cancer	1					3	3	4
Sarcoma	1		6	11	3		6	8
Significant Unique Analyses	27	12	25	29	5	7	117	82
Total Unique Analyses	443		737		269		954	

Supplementary Figure 1. The mRNA expression patterns of PEDF and TNFAIP6 in different types of human cancer**.** A. Transcriptional expression of PEDF in 20 different types of cancer diseases (ONCOMINE database)**.** B. Transcriptional expression of TNFAIP6 in 20 different types of cancer diseases (ONCOMINE database)**.**

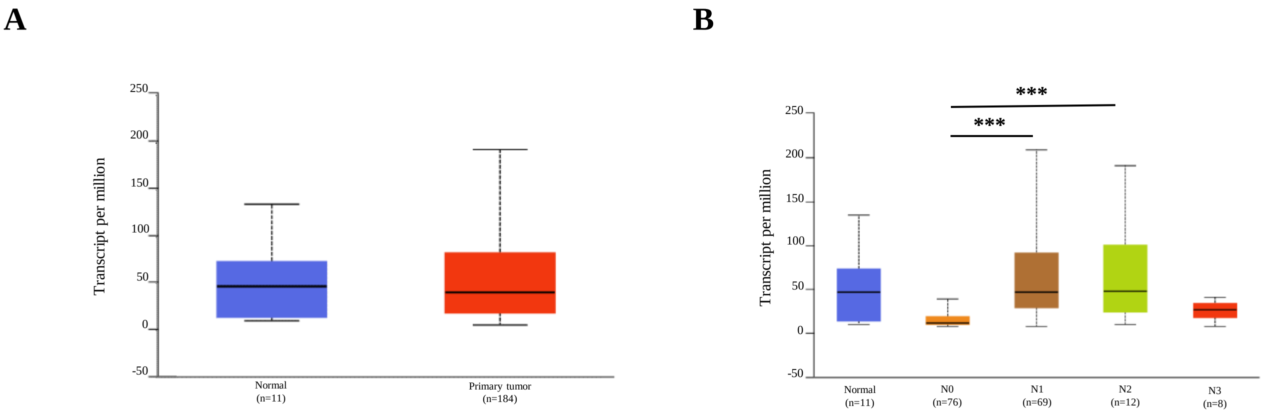

Supplementary Figure 2. Analysis of the level of PEDF mRNA expression in esophageal tissues based on the TCGA databases. A. The relative expression of PEDF in ESCC and normal tissues. B. The obvious correlation between N stages (metastatic lymph nodes) and PEDF expression levels. ***P < 0.001.

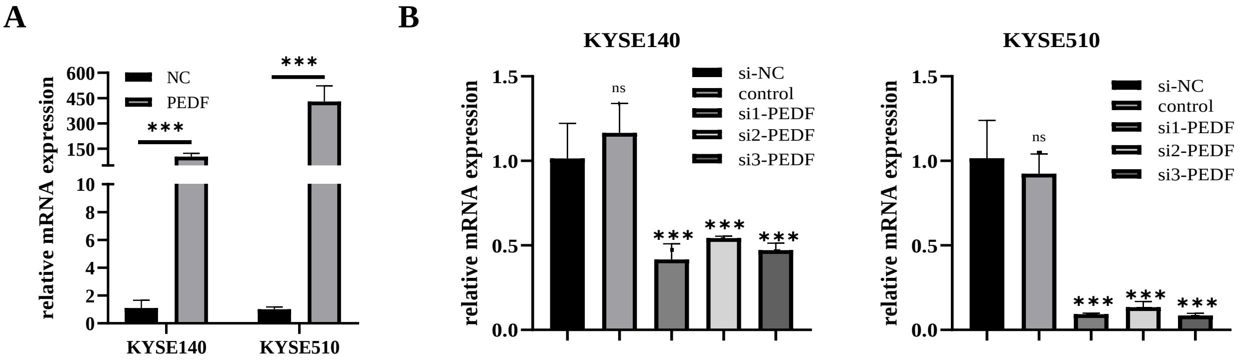

Supplementary Figure 3. The expression of PEDF mRNA in each cell line after overexpression or interference. A-B. Relative expression of PEDF mRNA was measured by qRT-PCR in ESCC cells transfected with PEDF overexpression plasmid and si-PEDF.
